# Targeted prebiotics alter the obese gut microbiome in humans

**DOI:** 10.1038/s41392-021-00758-2

**Published:** 2021-10-08

**Authors:** Junjun She, Chi Chun Wong, Jun Yu

**Affiliations:** 1 Department of General Surgery, The First Affiliated Hospital of Xi’an Jiaotong University, Xi’an, Shaanxi China; 2 Center for Gut Microbiome Research, Med-X Institute, The First Affiliated Hospital of Xi’an Jiao tong University, Xi’an, Shaanxi China; 3 Institute of Digestive Disease and Department of Medicine and Therapeutics, State Key Laboratory of Digestive Disease, Li Ka Shing Institute of Health Sciences, The Chinese University of Hong Kong, Hong Kong SAR, China

**Keywords:** Gastroenterology, Metabolic disorders

A recent study published in *Nature* by Delannoy-Bruno et al.^1^ described the role of formulated fibre snacks on the obese gut microbiome utilizing mouse-human co-clinical trial, and demonstrated that precise dietary fibre intervention directed at the obese gut microbiome modulates the host’s physiological state.

Epidemiological and observational studies^2^ have long linked a diet rich in fibre to protection against chronic diseases. Dietary fibre functions as a prebiotic that can be utilized by gut microbes. Given accumulating evidence showing roles of gut microbes in health and disease, there is renewed interest in harnessing fibre as a prebiotic to promote a healthy gut microbiome. Dietary fibres are safe, inexpensive, and readily incorporated into our diet. However, the development of evidenced-based fibre preparations that selectively induce the abundance of desirable gut microbes pose a huge challenge, as fibre and the gut microbiome are complex identities that remain poorly characterized. This is confounded by their dynamic interactions and inter- and intra-personal variations.

This study by Delannoy-Bruno et al.^1^ is part of a series of studies by the same group to develop microbiota-directed fibre formulations for targeting obese gut microbiome.^3,4^ In the first study by Ridaura et al., they established causality of dysregulated gut microbiome in obesity. Faecal transplantation of twin pairs (one lean and one obese) into germ-free mice led to increased adiposity only in those that received faecal transplants from the obese member, an effect that is rescued by invasion of specific members of *Bacteroidetes* from lean transplanted mice. In the second study by Patnode et al., they screened 34 food-grade fibres for targeted probiotics that selectively enrich beneficial *Bacteroidetes* species in germ-free mice fed a Western-style diet with high fat and low fibre.^4^ Integrative meta-proteomics and multi-species transposon mutagenesis showed that food fibres with compositionally distinct glycans specifically induce the outgrowth of members of *Bacteroidetes* expressing appropriate set(s) of glycan catabolic enzymes (carbohydrate-active enzymes, CAZymes). Armed with knowledge of the fibre preparations (pea, orange and barley) that best enrich *Bacteroidetes*, the authors designed a co-clinical trial involving germ-free mice reconstituted with obese microbiome and obese or overweight human patients to prove their hypothesis that targeted probiotics can rescue beneficial *Bacteroidetes* species, and ultimately alter host’s physiological state.

**Fig. 1:**
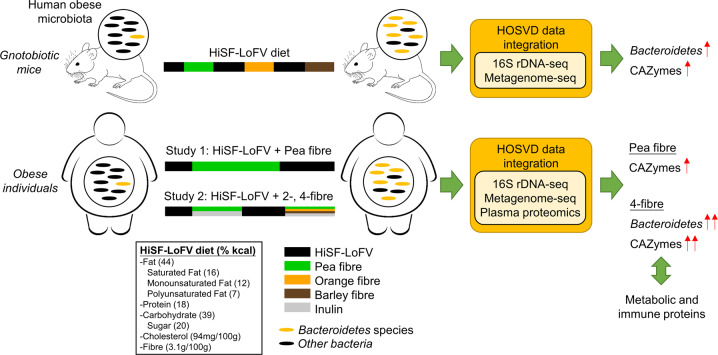
Co-clinical trial in gnotobiotic mice and obese individuals showed that targeted prebiotics alter the obese microbiome. In this study, the authors tested the effect of fibre supplementation regimens in (1) germ-free mice colonized with faecal microbiomes from obese individuals; and (2) obese or overweight subjects. Multiomic analysis demonstrated that specific fibre treatments lead to enriched beneficial *Bacteroidetes* species and is correlated with an altered physiological state

The authors evaluated the effects of three most promising fibres^1^ (pea, orange and barley fibres), with distinct glycan profiles, in germ-free mice colonized with faecal microbiomes from obese individuals and fed a high in saturated fats and low in fruits and vegetables (HiSF-LoFV) diet (Fig. 1). The authors used a machine learning approach (HOSVD) to integrate key features regarding the bacterial taxa (16s rDNA-seq), CAZymes and microbial metabolic pathways (Shotgun metagenome-seq). They successfully identified that treatment with fibres results in the enrichment of microbial genes encoding for CAZymes. Pea and orange fibres promoted both increased the abundances of genes involved in arabinan and galacturonan metabolism that reflects their similarity in glycan composition. Barley fibre decreased arabinose-metabolizing enzymes but promoted distinct β-glucanase activity. Importantly, these targeted fibres all led to the rise of *Bacteroidetes* species as the drivers of microbiome alterations. In correlation with altered metabolic activities, distinct *Bacteroidetes* responders to pea/orange (*B. thetaiotaomicron*, *B. vulgatus*) and barley (*B. ovatus*, *B. uniformis*, *B. xylanisolvens*) were found. The shift in microbial gene abundance is robust, inferring that dietary fibres confer a competitive advantage for select *Bacteroidetes* species.

To translate their findings in germ-free into humans, the authors recruited two pilot cohorts of obese or overweight individuals and subjected them to a HiSF-LoFV equivalent diet supplemented with different dietary fibres (Fig. 1). In Study 1, 12 participants were given HiSF-LoFV diet for 1 week prior to pea fibre snack supplementation for 3 weeks. Applying HOSVD, they demonstrated that the responses of microbial gene abundances were largely conserved between germ-free mice and humans after pea fibre supplementation, with enrichment of CAZymes in arabinose utilization pathway. In Study 2, a controlled diet study of 14 participants given HiSF-LoFV diet for 10 days, before addition of 2-fibre (pea + inulin) for 2 weeks. After a 10-day washout with HiSF-LoFV diet, HiSF-LoFV plus 4-fibre (pea + orange + barley + inulin) was given for 2 weeks. HOSVD revealed that showed that 4-fibre diet induced a greater spectrum of glycan-metabolizing CAZymes (*n* = 19) in gut microbiome compared to 2-fibre or pea fibre diet, consistent with the inclusion of structurally diverse fibres. 2- or 4-fibre diets produced significant increases in *Bacteroidetes* species (*B. uniformis*, *B. xylanisolvens*, *B. caccae*) consistent with that in germ-free mice. To corroborate the impact of fibre-directed gut microbiome on host physiology, they profiled host plasma proteomics (>1000 proteins) and devised a pipeline to identify the associations between plasma proteins and CAZymes responses to pea fibre, 2-fibre and 4-fibre snacks. For example, CAZymes enriched in 4-fibre snacks correlated with plasma proteins involved in glucose metabolism, coagulation & thrombosis, IGF/IGFBP and FGF signalling pathways. Expanding their analyses to include non-CAZymes correlated plasma proteins, the authors revealed that 4-fibre diet increased TYK2, CXCL1 and NXPH1, genes inversely associated with obesity, whilst suppressing the adipokine FGF2. Collectively, these results imply that targeted prebiotic diet impacts gut microbiomes, which were linked to metabolic and immune functions of the host.

The paper by Delannoy-Bruno et al. provided a framework for precision nutrition, whereby designer fibre snacks, acting as a prebiotic, could be tailored towards the enrichment of beneficial microbes in obese individuals. Utilizing multiomics and HOSVD, it builds a novel platform for evidence-based elucidation of diet–microbiome–host axis. This study offers mechanistic insights into how dietary fibre supplementation enriches for specific microbial genes that correlate with changes in the host’s plasma proteome. It also shows that the diet–microbiome–host axis is readily and robustly modifiable by targeted prebiotics. Nevertheless, this clinical trial is a short-term study, and it is unclear how resilient is the fibre-directed gut microbiome in modulating the host’s status, and if long-term fibre intervention may have undesirable consequences. The next step should involve long-term clinical studies which is designed to assess the durability of targeted prebiotics in restoring a healthy microbiome and to monitor more health-related outcomes in human subjects in longitudinal studies.
