# Correction: Targeted prebiotics alter the obese gut microbiome in humans

**DOI:** 10.1038/s41392-021-00871-2

**Published:** 2022-01-13

**Authors:** Junjun She, Chi Chun Wong, Jun Yu

**Affiliations:** 1 Department of General Surgery, The First Affiliated Hospital of Xi’an Jiaotong University, Xi’an, Shaanxi China; 2 Center for Gut Microbiome Research, Med-X Institute, The First Affiliated Hospital of Xi’an Jiao tong University, Xi’an, Shaanxi China; 3 Institute of Digestive Disease and Department of Medicine and Therapeutics, State Key Laboratory of Digestive Disease, Li Ka Shing Institute of Health Sciences, The Chinese University of Hong Kong, Hong Kong SAR, China

**Keywords:** Gastroenterology, Metabolic disorders

Correction to: *Signal Transduction and Targeted Therapy* 10.1038/s41392-021-00758-2, published online 8 October 2021

In the original version of this article^1^ given name of first author Junjun She was supplied and published incorrectly as Junyun She.

The original article has been corrected.
